# Discrepancies in the Registries of Diet vs Drug Trials

**DOI:** 10.1001/jamanetworkopen.2019.15360

**Published:** 2019-11-13

**Authors:** David S. Ludwig, Cara B. Ebbeling, Steven B. Heymsfield

**Affiliations:** 1 New Balance Foundation Obesity Prevention Center, Boston Children’s Hospital, Harvard Medical School, Boston, Massachusetts; 2 Metabolism & Body Composition Laboratory, Pennington Biomedical Research Center, Baton Rouge, Louisiana

## Abstract

This cross-sectional study examines discrepancies between registered protocols and subsequent publications for drug and diet trials whose findings were published in prominent clinical journals in the last decade.

## Introduction

ClinicalTrials.gov was established in 2000 in response to the Food and Drug Administration Modernization Act of 1997, which called for registration of trials of investigational new drugs for serious diseases. Subsequently, the scope of ClinicalTrials.gov expanded to all interventional studies, including diet trials. Presently, prospective trial registration is required by the National Institutes of Health for grant funding and many clinical journals for publication.^1^ Registration may reduce risk of bias from selective reporting and post hoc changes in design and analysis.^1,2^ Although a study^3^ of trials with ethics approval in Finland in 2007 identified numerous discrepancies between registered protocols and subsequent publications, the consistency of diet trial registration and reporting has not been well explored.

## Methods

This cross-sectional study compared the registries of drug and diet trials published in selected prominent clinical journals in the last decade. A literature search, conducted June 26, 2019, retrieved trials with obesity-related outcomes in 5 general medical journals and 1 nutrition journal with the highest impact factors for their fields (*The New England Journal of Medicine, JAMA, The BMJ, The Lancet, Annals of Internal Medicine,* and* The American Journal of Clinical Nutrition*). Results were limited to clinical trials in the 10-year period ending June 1, 2019. For drug trials, the search terms were “drug OR placebo OR pharmaceutical” with exclusion if the intervention did not focus on a specific drug (eg, dietary supplement, food extract). For diet trials, the search terms were “diet OR dietary” with exclusion if the intervention did not focus on a specific diet (eg, dietary supplement, food extract, dietary pattern such as meal skipping, or multicomponent intervention such as one including exercise). Additionally, trials in both categories were excluded if the article was not in the indicated journals (eg, *BMJ Open*) or was not an original randomized clinical trial; the primary registry was not in ClinicalTrials.gov; the primary outcome was not related to body weight, adiposity, or energy balance; or the primary outcome was measured in less than 28 days. For each included trial, we examined the current ClinicalTrials.gov registry, the history of changes at the ClinicalTrials.gov archive site, and the final published article, with reference to changes in or discrepancies involving the prespecified primary outcome. We calculated an odds ratio and 95% confidence interval for the number of diet vs drug trials with changes or discrepancies using SAS statistical software version 9.4 (SAS Institute). A 2-tailed *P* < .05 was considered statistically significant. The ethical review board of Boston Children’s Hospital does not require review of this type of study because no human participants were involved.

## Results

Our literature search retrieved 148 drug studies and 343 diet studies, from which 9 and 21, respectively, were included in our sample after applying exclusion criteria. As shown in Table 1 and Table 2, 2 drug trials (22%) and 18 diet trials (86%) had a substantive discrepancy from initial registration, typically involving a change in time frame of the primary outcome or the number of co–primary outcomes. The odds ratio for a discrepancy for diet vs drug trials was 21.0 (95% CI, 2.9-153.8; *P* = .002). Among diet trial registries, 2 appeared to have been post hoc and 1 was not available.

**Table 1.** Drug Registries Involving Obesity in Selected Major Medical Journals

PMID	ClinicalTrials.gov Identifier	Primary Outcome		Comment
		As Initially Registered	In Published Study	
30122305	NCT02453711	“Relative change in body weight (%) wk 0, wk 52”	As initially registered	
29945727	NCT02548585	“Percent change from baseline in MMT glucose AUC (up to 240 minutes post MMT) to end of treatment”; “change from baseline in body weight in kg to end of treatment”; both outcomes for “Cohort 4 only”	Primary outcome tested in phase 2a part of study, not dose escalation part	Dose escalation cohorts indicated by numbers (eg, cohort 4) in registry and letters (cohorts A-E) in published study
26840133	NCT01273584	“Birthweight centile (*z*-score) at birth”	As initially registered	Presented as median difference in published study
26284720	NCT01272232	“Change from baseline in body weight (fasting) wk 0, wk 56”; “proportion of subjects losing at least 5% of baseline body weight at 56 wk”; “proportion of subjects losing more than 10% of baseline body weight at 56 wk”	As initially registered	
26132939	NCT01272219	“Change from baseline in body weight (fasting) wk 0, wk 56”; “proportion of subjects losing at least 5% of baseline body weight at 56 wk”; “proportion of subjects losing more than 10% of baseline body weight at 56 wk”; “proportion of subjects with onset of type 2 diabetes at 160 wk”	As initially registered	Co–primary outcomes 1-3 presented in this published study; co–primary outcome 4, related to diabetes, published in *Lancet* 2017; 389:1399-1409; PMID: 28237263
21481449	NCT00553787	“Mean percent loss of baseline body weight and percent of subjects with at least 5% weight loss at 56 wk”	As initially registered	
20673995	NCT00532779	“The percentage of total body weight lost and the percentage of subjects who achieve a weight decrease of ≥5% at 56 wk”	As initially registered	
20647200	NCT00395135	“Proportion (%) of patients achieving ≥5% weight reduction at the end of the first year of treatment (wk 52)”; “proportion of patients maintaining ≥5% weight reduction at the end of year 2 (wk 104)”	5 Co–primary outcomes listed, including 1 with a threshold of 10% weight reduction	Primary outcomes amended in registry after publication (submitted January 4, 2013)
19853906	NCT00422058	“Body weight loss after 20 wk of treatment”	As initially registered	

Abbreviations: AUC, area under the curve; MMT, mixed-meal test; PMID, PubMed identification number.

**Table 2.** Diet Registries Involving Obesity in Selected Major Medical Journals

PMID	ClinicalTrials.gov Identifier	Primary Outcome		Comment
		As Initially Registered	In Published Study	
30429127	NCT02068885	“Total energy expenditure, assessed by indirect calorimetry using stable isotopes baseline through 20 wk weight loss maintenance”	Total energy expenditure from immediately after weight loss through 20 wk	Posted final analysis plan specifying the time immediately after weight loss as the baseline (submitted September 19, 2017)
29466592	NCT01826591	“Change from baseline in weight at 12 mo”; “baseline, 3 mo, 6 mo, 12 mo”	Change from baseline in weight at 12 mo	Interim points (3, 6 mo) not included; primary outcome amended in registry (submitted July 27, 2017)
28747328	NCT00938808	“Weight, No. of patients operated with knee alloplasty 1 y, 3 y”	Change in body weight after 3 y and the number of participants who received total knee alloplasty during the 3-y intervention	1-y data not included in the primary outcome (provided instead in supplement table)
28298396	NCT01066806	“Weight 5 y”	Percent change in body fat at 1 y	Primary outcome amended in registry (submitted November 3, 2011)
27903520	NCT01750021	“Changes in adipose tissue”; “CT, body composition, molecular analyses of adipose tissue”; “time frame: baseline and 3 months”	Study powered on “change of visceral fat mass measured by computed tomography”	Numerous outcomes presented as primary; time frame amended in registry (submitted January 5, 2014) to include 6 mo data; published study presents only 3 mo data
26075751	NCT01152359	“Body weight 48 wk”	As initially registered	Registry amended after publication to “percent change in body weight ” (submitted January 9, 2019)
25178568	NCT00609271	“Body weight”; “body composition”; both outcomes measured at “randomization, 3, 6, 12 mo”	Multiple anthropometric variables and disease risk factors	Study powered on body weight; authors state: “Because of the number of tests performed in the primary analyses, statistically significant results should be interpreted with caution”; registry amended after publication to include 16 discrete primary outcomes (submitted April 25, 2018)
24257725	NCT01195610	“Change in body weight “; “body fat percentage”; “dietary compliance level”; “components of metabolic syndrome”; all outcomes measured at 26 wk	Change in body weight from wk 0 to wk 26	Primary outcome amended in registry (submitted April 25, 2012); other initial primary outcomes presented as secondary outcomes in published study
23255569	NCT01068197	“Change in insulin sensitivity between the 2 dietary groups”; “change in BMI *z*-score between the 2 dietary groups”; both outcomes measured at 3-, 12-, and 24 mo	BMI *z*-score	Manuscript states “BMI *z* score ... was the predetermined primary efficacy variable used to assess the effectiveness of treatments”; insulin resistance (sensitivity) secondary outcome
22998340	NCT00893529	“Body weight 0, 6, 12, and 18 mo”	BMI *z*-score change from baseline to 18 mo	Primary outcome amended in registry to “body mass index *z*-score 0, 6, 12, and 18 months” (submitted February 4, 2011); interim points not included in calculation of primary outcome in published study
22998339	NCT00381160	“Body mass index”	BMI change through 2 y	Primary outcome amended in registry (submitted November 29, 2007); in the published study, 2-y data presented as primary, 1-y data as secondary
22743313	NCT00194428	“Body weight and body composition in overweight and obese person”	Multiple outcomes at 6 and 18 mo, none specified as primary	Registry amended to specify 6-mo but not 18-mo point (submitted January 3, 2008)
22301929	NCT01017783	“Weight change 0, 3, 6 mo”	As initially registered	Manuscript states “primary hypothesis … was that participants assigned to the beverage substitution groups would achieve greater weight loss at 6 mo”; both 3 and 6 mo data presented in published study
22205311	NCT00777647	“Body weight; MR spectroscopy; MRI; DEXA scan 6 mo”	Multiple anthropometric variables and disease risk factors	No outcome specified as primary in published study
21715516	NCT01266330	“Body weight”; “body composition DEXA assessment”; “oxidative stress plasma malonaldehyde, 8-isoprostane F2α and oxidized LDL”; “inflammatory stress plasma L-6 [*sic*], IL-15, MCP, CRP, adiponectin and TNF-α”; all measured at 12 weeks	“Oxidative and inflammatory biomarkers” at 0, 1, 4, and 12 wk	Published study states power calculation based on preliminary data involving CRP; body weight and composition indicated as secondary outcomes; registered after participants recruited (initial enrollment indicated as “actual”)
21105792	NCT00390637	“For adults: body weight loss maintained, body composition, proportion of subjects maintaining >0, 5 and 10% weight loss, and dropout rate”	Body weight loss maintenance after 6 mo	Registry amended to specify 6-mo point (submitted November 3, 2007) and further amended after publication to specify body weight loss maintenance (submitted May 29, 2017)
20962162	NCT00364403	“Birth weight as assessed by *z*-scores”	As initially registered	
20679559	NCT00143936	None listed	Weight loss at 2 y	Primary outcome amended in registry (submitted January 3, 2008)
20647285	NCT00124553	“BMI”; “glycated hemoglobin”; “triglycerides”; “dietary intake as measured by 3-day weighed diet records”; all measured at 6 mo	Glycated hemoglobin at 6 mo	Several initially listed co–primary outcomes included as secondary outcomes in published study
19889829	NCT00686426	“Body weight change”; “body fat change”; both measured at 6 mo	Numerous outcomes with a focus on oxidative and inflammatory stress	Major discrepancies involving participant number (registry 338; published study 20), intervention (registry after weight loss; published study before weight loss) and other features; study completed July 2007, registered May 2008
19793858	NCT00625236	Registry number indicated in published study not found in ClinicalTrials.gov	Multiple anthropometric variables and cardiovascular disease risk factors, none specified as primary	Coded as a discrepancy in the primary outcome owing to lack of registration

Abbreviations: BMI, body mass index; CRP, C-reactive protein; CT, computed tomography; DEXA, dual-energy X-ray absorptiometry; LDL, low-density lipoprotein cholesterol; MCP, monocyte chemoattractant protein 1; MMT, mixed-meal test; MR, magnetic resonance; MRI, magnetic resonance imaging; PMID, PubMed identification number; TNF-α, tumor necrosis factor α.

## Discussion

This study found that, among top-rated clinical journals, most diet trials would not satisfy an essential criterion for prospective registration, as judged by standards inferred from drug trials. This problem extends beyond low registration rates in behavioral research,^4^ as registration for only 1 diet trial was missing.

Limitations of this study include a focus on just 1 (though central) aspect of registration and extrapolation of the findings to the general literature. We did not examine registry data involving interventions, participant number (anticipated vs achieved), or statistical treatments. Although our findings derive from a small subset of published trials, the highlighted problems are likely general to the field, in light of the highly selective nature of the journals examined. Furthermore, we did not control for measures of trial quality because these could be considered inherently related to, rather than confounders of, the observed association.

Problems with diet trial registries may arise from the their greater heterogeneity and lower budgets vs drug studies and the inadequacy of infrastructural support for nutrition research.^5^ Ultimately, high-quality diet trials of the type needed to develop effective prevention and treatment for chronic disease will require substantial investment of financial and personnel resources from the National Institutes of Health and philanthropy. More immediate and specific remedies for the deficiencies of diet trial registries include (1) posting a detailed final statistical analysis plan before unmasking random group assignments or beginning data analyses as a minimum quality criterion; (2) declaring substantive changes to the original registry in the main manuscript (rather than infrequently read online supplementary material); and (3) creating specialized registries for diet (and possibly other behavioral) trials to reflect their special challenges beyond those of drug trials (for which current registries were originally intended).
